# Effect of Cation Ordering on the Performance and Chemical Stability of Layered Double Perovskite Cathodes

**DOI:** 10.3390/ma11020196

**Published:** 2018-01-26

**Authors:** Carlos Bernuy-Lopez, Laura Rioja-Monllor, Takashi Nakamura, Sandrine Ricote, Ryan O’Hayre, Koji Amezawa, Mari-Ann Einarsrud, Tor Grande

**Affiliations:** 1 Department of Material Science and Engineering, NTNU Norwegian University of Science and Technology, NO-7491 Trondheim, Norway; laura-rioja-monllor@ntnu.no (L.R.-M.); mari-ann.einarsrud@ntnu.no (M.-A.E.); tor.grande@ntnu.no (T.G.); 2 Institute of Multidisciplinary Research for Advanced Materials, Tohoku University, 2-1-1 Katahira Aoba-ku, Sendai 980-8577, Japan; t-naka@tagen.tohoku.ac.jp (T.N.); amezawa@tagen.tohoku.ac.jp (K.A.); 3 Department of Mechanical Engineering, Colorado School of Mines, Golden, CO 80401, USA; sricote@mines.edu; 4 Department of Metallurgical and Materials Engineering, Colorado School of Mines, 1500 Illinois St., Golden, CO 80401, USA; rohayre@mines.edu

**Keywords:** proton ceramic fuel cells (PCFC), cathode, layered double perovskite

## Abstract

The effect of A-site cation ordering on the cathode performance and chemical stability of A-site cation ordered LaBaCo_2_O_5+δ_ and disordered La_0.5_Ba_0.5_CoO_3−δ_ materials are reported. Symmetric half-cells with a proton-conducting BaZr_0.9_Y_0.1_O_3−δ_ electrolyte were prepared by ceramic processing, and good chemical compatibility of the materials was demonstrated. Both A-site ordered LaBaCo_2_O_5+δ_ and A-site disordered La_0.5_Ba_0.5_CoO_3−δ_ yield excellent cathode performance with Area Specific Resistances as low as 7.4 and 11.5 Ω·cm^2^ at 400 °C and 0.16 and 0.32 Ω·cm^2^ at 600 °C in 3% humidified synthetic air respectively. The oxygen vacancy concentration, electrical conductivity, basicity of cations and crystal structure were evaluated to rationalize the electrochemical performance of the two materials. The combination of high-basicity elements and high electrical conductivity as well as sufficient oxygen vacancy concentration explains the excellent performance of both LaBaCo_2_O_5+δ_ and La_0.5_Ba_0.5_CoO_3−δ_ materials at high temperatures. At lower temperatures, oxygen-deficiency in both materials is greatly reduced, leading to decreased performance despite the high basicity and electrical conductivity. A-site cation ordering leads to a higher oxygen vacancy concentration, which explains the better performance of LaBaCo_2_O_5+δ_. Finally, the more pronounced oxygen deficiency of the cation ordered polymorph and the lower chemical stability at reducing conditions were confirmed by coulometric titration.

## 1. Introduction

Proton ceramic fuel cells (PCFC) can potentially overcome some of the challenges currently limiting the commercial application of conventional solid oxide fuel cells (SOFCs) [1,2,3,4]. The main difference between conventional SOFCs and PCFCs is the electrolyte material. While SOFCs employ oxide-ion conducting electrolytes, PCFCs make use of proton-conducting electrolytes instead. As the activation energy for protons is lower than for oxide ions, PCFCs can operate at lower temperatures than conventional SOFCs, i.e., 400–600 °C [5] vs. 700–900 °C. However, one of the main issues confronting PCFCs is the lack of high performance cathode materials [1]. A suitable cathode material for PCFCs must facilitate the reduction of oxygen to water by reacting with protons that diffuse through the proton-conducting electrolyte. An ideal high-performance cathode material should combine the conduction of electrons (or holes), oxide ions and protons at the same time [6,7]. Equally important, the material must be chemically stable at the operating conditions.

Mixed oxide-ion- and electron-conducting materials with a perovskite structure are the most promising cathodes so far. Unfortunately, the best cathode materials for conventional SOFCs, such as La_0.6_Sr_0.4_Co_0.2_Fe_0.8_O_3−δ_ (LSCF) [8] and Ba_0.5_Sr_0.5_Co_0.8_Fe_0.2_O_3−δ_ (BSCF) [8], do not seem to present proton conductivity despite their good electronic and oxide-ion conductivities. Several key parameters in perovskite oxides can be tuned to enhance proton conductivity while ensuring good electronic and oxide-ion-conductivity: these include crystallographic structure, oxygen vacancy concentration, electrical conductivity and basicity [6,9,10,11]. Regarding the crystallographic structure it is established that cubic structures favor both ionic and electronic conductivity [5]. In respect to the oxygen vacancy concentration, high oxygen vacancy concentration can enhance proton conductivity at intermediate temperatures as a result of the Wagner hydration reaction [12,13]:

$$H2O+V_{O}^{••}+O_{O}^{x}↔{2\mathrm{OH}}_{O}^{•}$$

In addition, electrical conductivity above 1 S/cm is required for adequate cathode performance [3]. Finally, high Ba content is desirable as it leads to higher oxide basicity and thereby a greater degree of protonation of the oxygen vacancies [13,14].

Layered double perovskite materials with the general formula LnBaM_2_O_5+δ_ (Ln = lanthanide or Y; M = transition metal) have been studied as potential electrodes for both PCFC [15,16,17,18] and SOFC due to their outstanding mixed electronic and oxide-ion conductivities [19,20]. Ln and Ba occupy the A-site in this double perovskite AA′B_2_O_6_-type crystal structure, while M occupies the B-site. A-site cation ordering is adopted due to the large difference of size between Ba and Ln with LnO and BaO layers in dodecahedral coordination separated with MO_6_ layers in octahedral coordination [21]. Cation ordering results in a decrease of symmetry. Layered double perovskite materials can adopt large concentrations of oxygen vacancies and depending on the size of Ln and the nature of M, the material will adopt either a tetragonal or an orthorhombic symmetry as vacancy ordering occurs [22]. A-site cation ordering is reported to be beneficial for oxide-ion conductivity [23] while the ordering of the oxygen vacancies is detrimental [24].

Strandbakke et al. have reported outstanding performance for the layered double perovskite La_0.2_Gd_0.8_BaCo_2_O_5+δ_ [16] as a PCFC cathode with Area Specific Resistances (ASR) as low as 6 Ω·cm^2^ at 400 °C and 3% H_2_O in air. The large oxygen vacancy concentration adopted by the layered double perovskite seems to favor proton incorporation and sufficient proton conductivity. The performance is comparable to that of mixed electron/proton conducting single perovskite materials such as BaCo_0.4_Fe_0.4_Zr_0.1_Y_0.1_O_3−δ_ (BCFZY) [3] (ASR = 10 Ω·cm^2^ at 400 °C in 3% humidified air), although the benefits of the layered double perovskite crystal structure are still unclear.

LaBaCo_2_O_5+δ_ represents an interesting model system to study the influence of ordering effects on the performance of PCFC cathode materials. In addition to the A-site ordered phase, this material can also adopt an A-site cation disordered cubic structure, represented as La_0.5_Ba_0.5_CoO_3−δ_, due to the larger size of La compared with other Ln elements. In our recent work [25], we demonstrated that the ordered LaBaCo_2_O_5+δ_ phase is a metastable variant of the A-site cation disordered phase, La_0.5_Ba_0.5_CoO_3−δ_. Several authors have studied the effects of A-site cation ordering on the performance of LaBaCo_2_O_5+δ_ and La_0.5_Ba_0.5_CoO_3−δ_ for SOFC application [26,27,28], although it has yet to be studied for PCFC application. Both the ordered and disordered variants demonstrate low polarization resistances at temperatures as low as 600 °C (<0.2 Ω·cm^2^) due to the excellent mixed conducting (electron hole and oxide-ion) nature of the material. In addition, Garces et al. have studied the influence of the A-site cation ordering on the mixed electronic and oxide-ion conducting properties in this system [29,30]. They obtained a noticeable improvement of performance with A-site cation ordering (0.35 Ω·cm^2^ for La_0.5_Ba_0.5_CoO_3−δ_ vs. 0.12 Ω·cm^2^ for LaBaCo_2_O_5+δ_ at 600 °C in air).

In this work, we examine proton conducting electrolyte supported symmetric cells employing both A-site cation ordered and disordered materials (LaBaCo_2_O_5+δ_ and La_0.5_Ba_0.5_CoO_3−δ_) to evaluate the effect of A-site cation ordering on performance for PCFC cathode applications. Cathode performance is evaluated by impedance spectroscopy and the results are analyzed with respect to crystal structure, basicity, oxygen content and ordering, and electrical conductivity. Finally, chemical compatibility between the cathode and the electrolyte is reported as well as chemical stability and oxygen deficiency by coulometric titration.

## 2. Experimental

### 2.1. Preparation of the Materials

La_0.5_Ba_0.5_CoO_3−δ_ was obtained by spray pyrolysis (Cerpotech AS, Tiller, Norway, purity > 99%) of nitrate precursors as described elsewhere [25]. The as-sprayed powders were calcined at 1100 °C for 12 h in air in order to obtain a single pure phase. LaBaCo_2_O_5+δ_ was obtained by calcining La_0.5_Ba_0.5_CoO_3−δ_ in slightly lower pO_2_ (N_2_ atmosphere, pO_2_ ~ 10^−4^ atm) at 1100 °C for 12 h. Phase purity for all materials were determined using a Bruker D8 Advance DaVinci X-ray diffractometer (Trondheim, Norway).

BaZr_0.9_Y_0.1_O_3−δ_ (BZY10) powder was prepared by spray pyrolysis (Cerpotech AS, Tiller, Norway, purity > 99%) of nitrate precursors as described elsewhere [31]. Green pellets of 20 mm diameter were prepared and sintered at 1650 °C for 10 h as described by Sazinas et al. [32]. Prior to electrode deposition, the pellets were polished with SiC paper and washed with ethanol.

Electrode slurries of LaBaCo_2_O_5+δ_ and La_0.5_Ba_0.5_CoO_3−δ_ were prepared by mixing 5 g of each powder with 1 g dispersant (20 wt % solsperse 28,000 (Lubrizol, Wickliffe, OH, USA) dissolved in terpineol), and 0.3 g binder (5 wt % V-006 (Heraeus, Hanau, Germany) dissolved in terpineol).

Electrolyte-supported symmetric cells for LaBaCo_2_O_5+δ_ and La_0.5_Ba_0.5_CoO_3−δ_ were produced by screen painting the corresponding slurries on both sides of a dense BZY10 pellet (geometrical density >90%). The thickness of the BZY10 electrolyte was about 800 µm after polishing and electrode thicknesses were ~20–25 µm. Thickness was checked by scanning electron microscopy (SEM). SEM images were captured on a field emission gun SEM (Zeiss Ultra 55, Limited Edition, Oberkochen, Germany). The symmetric cells of both LaBaCo_2_O_5+δ_ and La_0.5_Ba_0.5_CoO_3−δ_ materials were fired at 600 °C for 2 h in ambient air to form porous cathode layers. Gold paste (Fuel Cell Materials) was applied onto the cathodes for current collection followed by in-situ curing. Pt wires were employed as conducting wires.

### 2.2. Electrochemical Characterization

Symmetric cells were characterized by electrochemical impedance spectroscopy (EIS) in dry and moist (pH_2_O = 0.03 atm) synthetic air and N_2_ from 600 to 400 °C, at temperature intervals of 50 °C (with a cooling rate of 1 °C/min and 8 h dwell before each measurement) using a ProboStat™ (NorECs AS, Oslo, Norway) set-up and an Alpha A (Novocontrol Technologies, Montabaur, Germany) impedance analyzer. The signal amplitude was 50 mV under open circuit voltage (OCV) in the 10^−2^–10^6^ Hz frequency range. The 3% humidification was achieved by bubbling the gases through distilled water at 25 °C. The equivalent circuit fitting and analysis of the impedance data were carried out using Zview Software v3.5.

### 2.3. Oxygen Deficiency and Chemical Stability

Compatibility tests between the electrode and the electrolyte materials were performed by mixing together about 1 g each of both materials in an agar mortar for 15 min. Pellets of 15 mm diameter were fabricated and exposed to different thermal treatments: 1000 °C, 1100 °C and 1200 °C for 72 h at each temperature.

High Temperature X-ray diffraction (HT-XRD) measurements were performed using a Bruker D8 Advance diffractometer equipped with an MRI TCP20 high temperature camera (Sendai, Japan). A Pt strip-type resistive heater functioned as the sample support. XRD patterns (20–85°, about 30 min collection time) were recorded from 600 to 1200 °C in air, at 100 °C intervals. An S-type thermocouple was used for temperature determination using the radiant heater. The heating rate and dwell time before data collection were 0.1 °C/s and 10 min respectively

Finally, coulometric titration of both LaBaCo_2_O_5+δ_ and La_0.5_Ba_0.5_CoO_3−δ_ materials was performed to determine the oxygen content and the chemical stability of these materials below 10^−4^ bar. The details of the experiment and the set-up are given elsewhere [33,34].

## 3. Results

### 3.1. Microstructure of the Symmetric Cells

**Figure 1:**
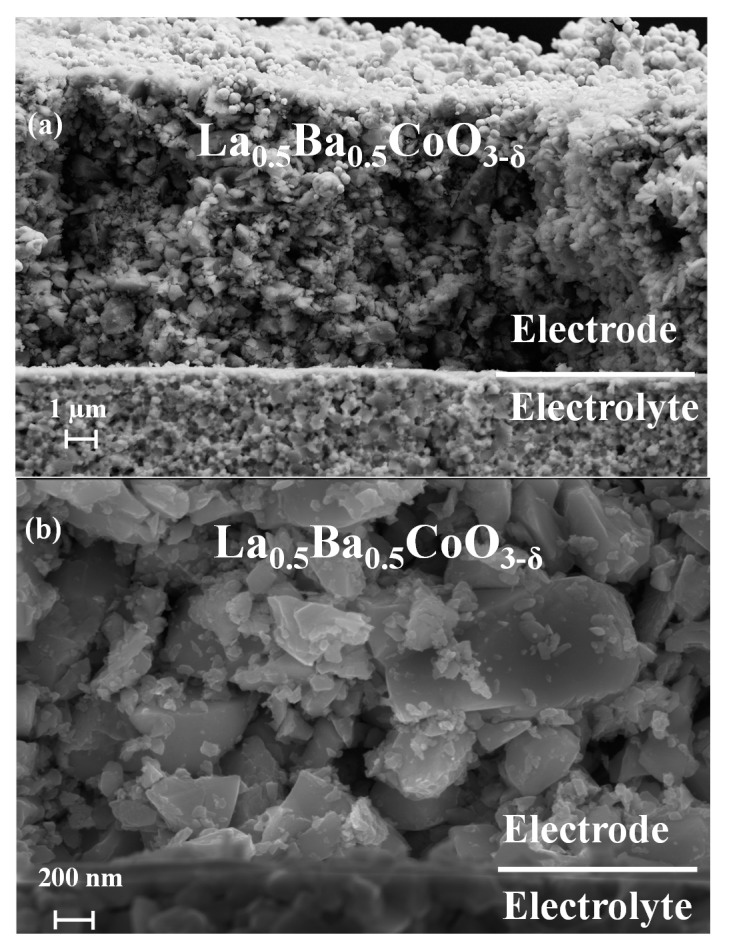
Scanning electron microscope pictures of pristine electrolyte supported symmetric cells of non-polished and cracked cross sections samples for La_0.5_Ba_0.5_CoO_3−δ_ at low (**a**) and high magnification (**b**).

X-ray diffraction of the two materials, as reported in our previous work [25], established the phase purity and crystal structure: cubic for La_0.5_Ba_0.5_CoO_3−δ_ and tetragonal for LaBaCo_2_O_5+δ_. Figure 1 provides representative low and high-magnification SEM images of a La_0.5_Ba_0.5_CoO_3−δ_ symmetric cell. Despite the low preparation temperature of the symmetric cells, sufficient adherence to the electrolyte was obtained. Higher processing temperatures lead to delamination and poor adherence of the electrolyte. Electrode thickness of about 20 µm and average grain size below ~1 µm are observed. LaBaCo_2_O_5+δ_ shows similar microstructure as shown in Figure S1.

### 3.2. Electrochemical Performance

**Figure 2:**
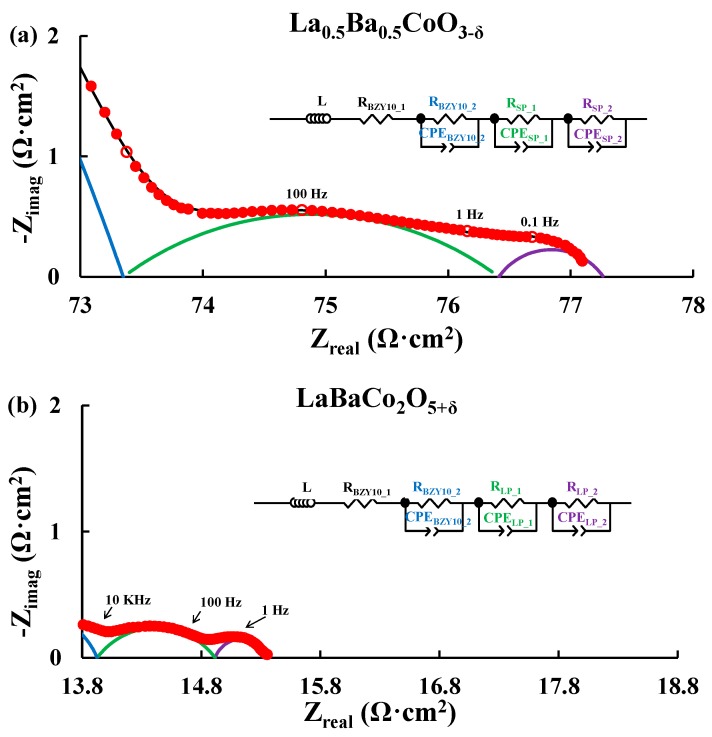
Nyquist plots for La_0.5_Ba_0.5_CoO_3−δ_ (**a**) and LaBaCo_2_O_5+δ_ (**b**) recorded at 500 °C in 3% H_2_O synthetic air. The red filled circles correspond to the experimental data with selected frequencies (10 KHz, 100 Hz, 1 Hz and 0.1 Hz) shown as open red circles. The equivalent circuit used to fit the data (black line) and Area Specific Resistance (ASR) are shown. The blue semicircle is the element assigned to the electrolyte and both the green and violet semicircles are the elements assigned to the electrode. The data are truncated for clarity.

Figure 2 depicts typical Nyquist plots obtained for symmetric cells of the A-site cation disordered La_0.5_Ba_0.5_CoO_3−δ_ and A-site cation ordered LaBaCo_2_O_5+δ_ materials in moist synthetic air at 500 °C. Both A-site cation disordered and ordered materials present similar Nyquist plots for all temperatures as illustrated in Figure 2. Two main contributions coming from the electrolyte and the electrode are observed. The equivalent circuit model used to fit the data is LR (RQ)(RQ)(RQ), where L, R and Q are inductance, resistance and constant phase element respectively. The resistor (R_BZY10_1_) and the first RQ element (R_BZY10_2_ and CPE_BZY10_2_, blue semicircle) are assigned to the electrolyte of the symmetric cells. The two other RQ elements (i.e., R_SP_1_, CPE_SP_1_, R_SP_2_, CPE_SP_2_, green and violet semicircles respectively for La_0.5_Ba_0.5_CoO_3−δ_) correspond to the electrode response of the cells. The assignment of these electrochemical processes was carried out by evaluating the pseudocapacitance of the RQ elements (Table S1 for both La_0.5_Ba_0.5_CoO_3−δ_ and LaBaCo_2_O_5+δ_) obtained with the electrochemical model: i.e., La_0.5_Ba_0.5_CoO_3−δ_; ~10^−10^ F/cm^2^ for the first RQ element, assigned as the response of the electrolyte [8,9,10,35,36,37,38,39,40,41]; ~10^−4^ F/cm^2^ and 10^−2^ F/cm^2^ for the other two RQ elements, assigned as the response of the electrode [8,9,10,35,36,37,38,39,40,41].

**Figure 3:**
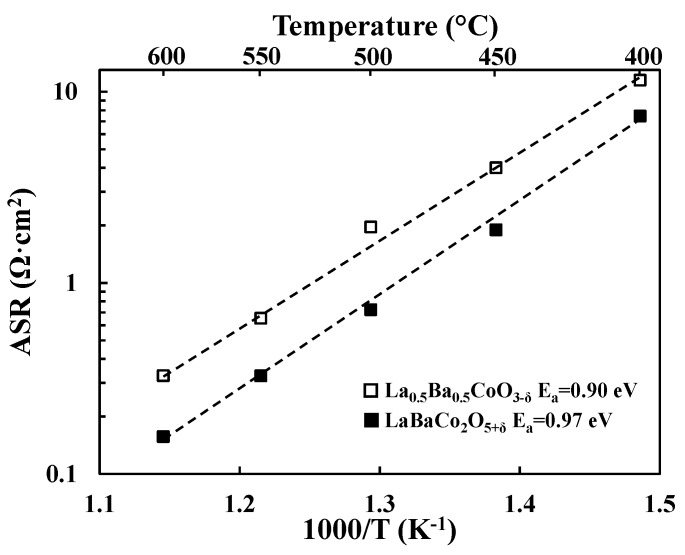
Area Specific Resistances (ASR, Ω·cm^2^) as a function of temperature for the single perovskite La_0.5_Ba_0.5_CoO_3−δ_ and the layered double perovskite LaBaCo_2_O_5+δ_ materials studied in this work in moist air. The lines represent the slope used to calculate activation energies (E_a_).

Total cathode Area Specific Resistances (ASRs) were obtained by dividing the sum of the electrode resistances (i.e., R_SP_1_ and R_SP_2_ in Figure 2 for La_0.5_Ba_0.5_CoO_3−δ_) by two. Division by a factor of two accounts for the fact that the combined contribution from two cathode electrode responses are measured in symmetric cell testing. Resulting cathode ASR values of both LaBaCo_2_O_5+δ_ and La_0.5_Ba_0.5_CoO_3−δ_ materials are shown in Figure 3 in 3% moist synthetic air. Both LaBaCo_2_O_5+δ_ and La_0.5_Ba_0.5_CoO_3−δ_ materials exhibit excellent performance in the temperature range 400–600 °C. Despite the similar microstructure of both materials, the A-site cation ordered material LaBaCo_2_O_5+δ_ gives a better performance than the A-site disordered La_0.5_Ba_0.5_CoO_3−δ_ material. Cathode ASR values for LaBaCo_2_O_5+δ_ at 600 and 400 °C are 0.15 and 7.4 Ω·cm^2^, respectively. The corresponding values for La_0.5_Ba_0.5_CoO_3−δ_ at 600 and 400 °C are 0.32 and 11.5 Ω·cm^2^, respectively. Activation energies are also given in Figure 3 for both La_0.5_Ba_0.5_CoO_3−δ_ and LaBaCo_2_O_5+δ_ materials, with higher E_a_ for A-site cation ordered LaBaCo_2_O_5+δ_.

**Figure 4:**
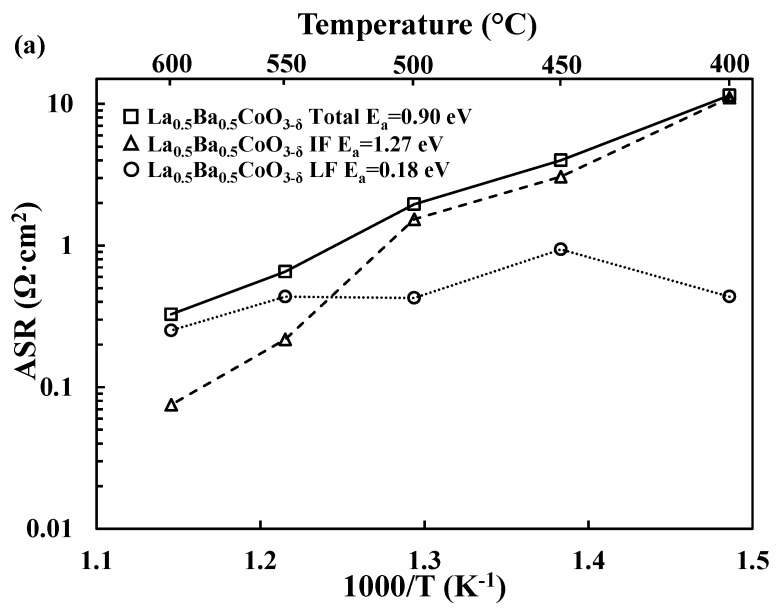

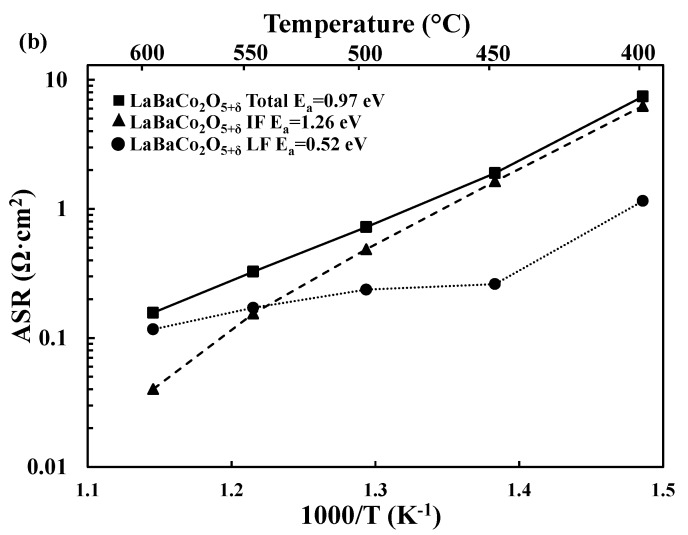
(**a**) Deconvolution of the Area Specific Resistance (ASR, Ω·cm^2^) as a function of temperature into the intermediate (IF) and low frequency (LF) processes for La_0.5_Ba_0.5_CoO_3−δ_ (**a**) and LaBaCo_2_O_5+δ_ (**b**) together with the activation energies.

For each material, the total electrode response can be deconvoluted in two main processes: an intermediate/middle frequency (R_SP_1_CPE_SP_1_) process and a low frequency process (R_SP_2_CPE_SP_2_). The intermediate frequency (MF) process exhibits lower pseudocapacitances than the low frequency (LF) process (~10^−4^ F/cm^2^ vs. 10^−2^ F/cm^2^). The deconvolution of the electrochemical data for both LaBaCo_2_O_5+δ_ and La_0.5_Ba_0.5_CoO_3−δ_ materials is shown in Figure 4. Similar trends are observed for both materials: below 550 °C, the LF process appears to be rate limiting, while the MF process becomes limiting at higher temperatures. Further investigation, e.g., pO_2_- and pH_2_O-dependent experiments would be needed to further assign these MF and LF processes to specific electrochemical reactions taking place in the electrode [39].

**Figure 5:**
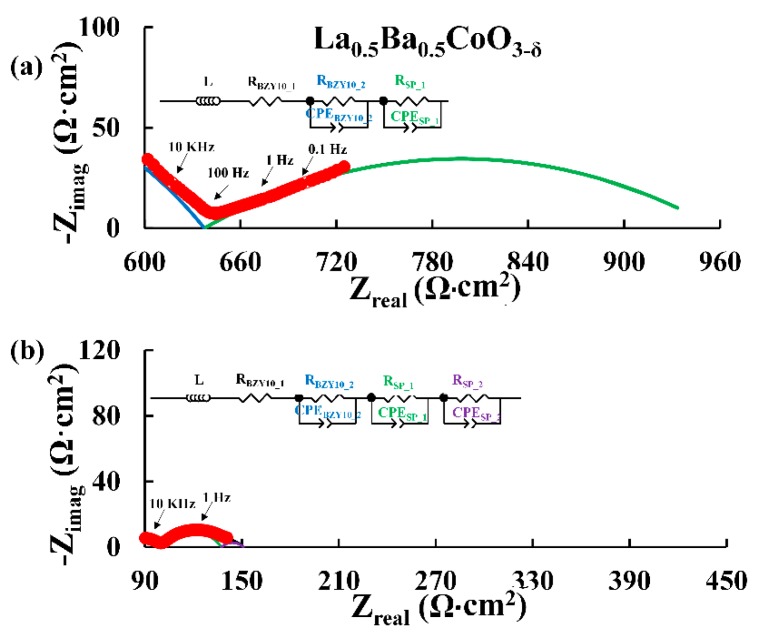
Nyquist plots for La_0.5_Ba_0.5_CoO_3−δ_ recorded at 500 °C in dry N_2_ (**a**) and 3% H_2_O N_2_ (**b**). The red filled circles are the experimental data with selected frequencies (10 KHz, 100 Hz, 1 Hz and 0.1 Hz) shown as open red circles. The equivalent circuit used to fit the data (black line) and the Area Specific Resistances (ASR) are shown. The blue semicircle is the element assigned to the electrolyte and both the green and violet semicircles are the elements assigned to the electrode. The data are truncated for clarity.

**Figure 6:**
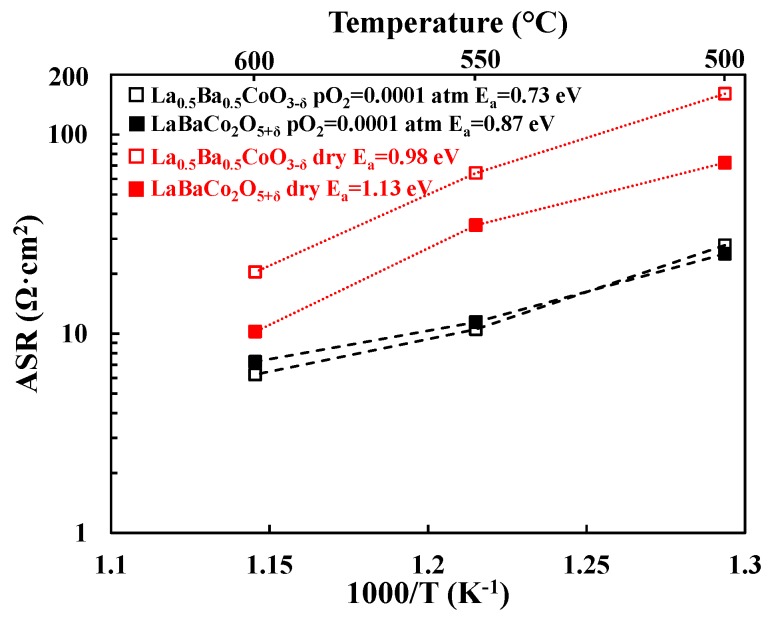
Area Specific Resistance (ASR, Ω·cm^2^) as a function of temperature for the La_0.5_Ba_0.5_CoO_3−δ_ and LaBaCo_2_O_5+δ_ materials in dry (red symbols) and moist N_2_ atmosphere (pO_2_ = 0.0001 atm). Lines are guides for the eye.

Figure 5 shows the Nyquist plots for La_0.5_Ba_0.5_CoO_3−δ_ in N_2_ atmosphere at 500 °C in dry (Figure 5a) and moist conditions (Figure 5b). ASR values for the electrode contribution extracted from these data are represented in Figure 6. It is found that the ASR decreases in N_2_ when the atmosphere is humidified. It can also be observed that A-site cation ordering does not give any improvement in the ASR as both A-site cation ordered and disordered materials lead to the same performance in moist conditions at low pO_2_.

### 3.3. Chemical Stability

**Figure 7:**
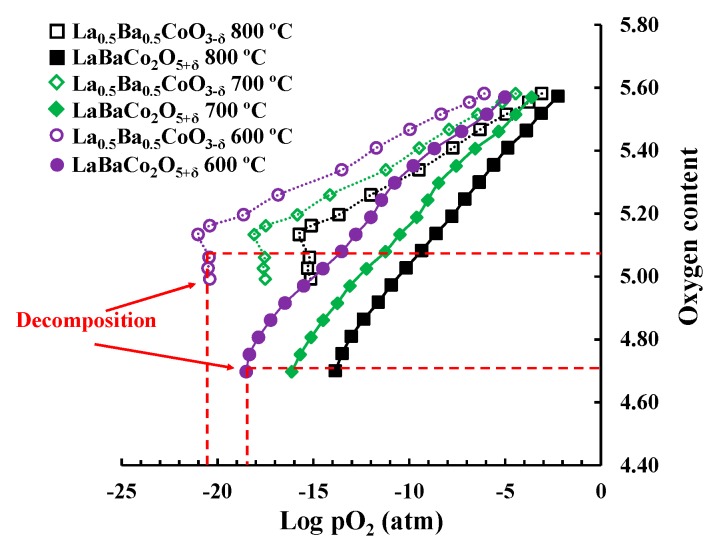
Coulometric titration for La_0.5_Ba_0.5_CoO_3−δ_ and LaBaCo_2_O_5+δ_ at 600, 700 and 800 °C. The red dotted lines show the decomposition of both materials at the indicated oxygen content and pO_2_ at 600 °C.

The chemical potential of oxygen and stability of the two polymorphs in reducing conditions were investigated by coulometric titration and the results are shown in Figure 7. The oxygen deficiency increases with decreasing oxygen partial pressure as expected, but the slope is significantly different for the two polymorphs. The difference in slope demonstrates the superior stability of Co in a higher oxidation state in La_0.5_Ba_0.5_CoO_3−δ_ relative to LaBaCo_2_O_5+δ_. This is further confirmed by the onset of decomposition (vertical relationship of stoichiometry versus pO_2_) of LaBaCo_2_O_5+δ_ at a higher pO_2_ relative to La_0.5_Ba_0.5_CoO_3−δ_ at constant temperature. Moreover, the coulometric data also proves that the cation-ordered phase tolerates a higher oxygen deficiency before decomposition although this could be an effect of the kinetics of the decomposition reaction.

**Figure 8:**
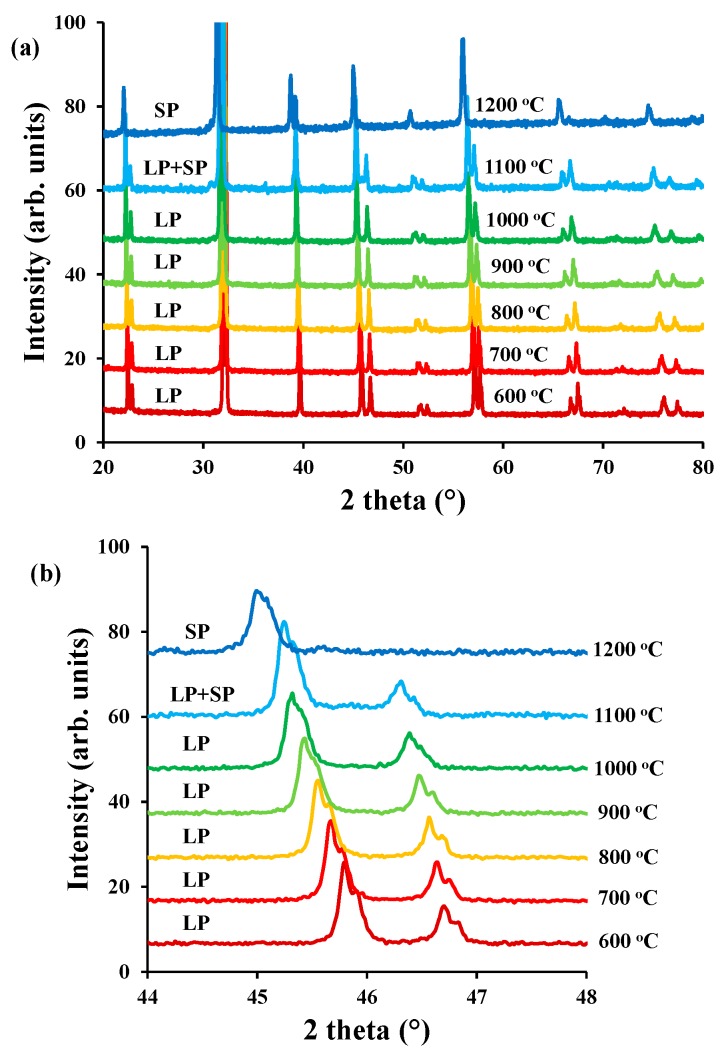
(**a**) High temperature X-Ray diffractogram for LaBaCo_2_O_5+δ_ between 600–1200 °C and 20–80° in air; (**b**) Inset between 44–48° the transformation from LaBaCo_2_O_5+δ_ (LP) to La_0.5_Ba_0.5_CoO_3−δ_ (SP) is observed by the disappearance of the peak splitting.

The thermal stability of LaBaCo_2_O_5+δ_ in air was studied by high temperature X-ray diffraction and the diffraction patterns are shown in Figure 8. Only thermal expansion of LaBaCo_2_O_5+δ_ was observed up to 1100 °C. These data are consistent with our previous study [25] where LaBaCo_2_O_5+δ_ was shown to remain tetragonal at high temperature with a P4/mmm as space group. This means that the material remains A-site ordered at the studied temperature range (RT-800 °C). However, Figure 8 shows that LaBaCo_2_O_5+δ_ starts to transform into La_0.5_Ba_0.5_CoO_3−δ_ at 1100 °C, with complete disappearance of the splitting of the Bragg reflections due to the loss of A-site cation ordering at 1200 °C (Figure 8b). This means that there is a phase transition from tetragonal P4/mmm structure of LaBaCo_2_O_5+δ_ to cubic $P\bar{3}mmm$ structure of La_0.5_Ba_0.5_CoO_3−δ_ close to 1100 °C in air.

Finally, a good compatibility between both LaBaCo_2_O_5+δ_ and La_0.5_Ba_0.5_CoO_3−δ_ materials and BZY10 electrolyte material is demonstrated by X-ray diffraction of powder mixtures annealed at different temperatures (Figure S2). Temperatures close to 1200 °C for 72 h are required to initiate (minor) secondary phase formation in a powder mixture of the two materials with the electrolyte. Both PCFC operation temperatures (400–600 °C) and electrode sintering temperature (600 °C) are well below the temperature where cathode/electrolyte reactions are observed to initiate.

## 4. Discussion

### 4.1. Comparison with Literature

**Figure 9:**
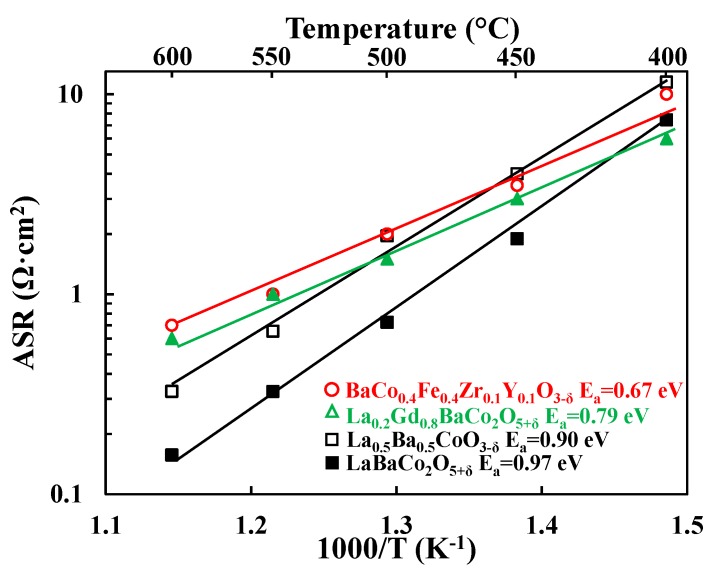
Area Specific Resistances (ASR, Ω·cm^2^) as a function of temperature for the single perovskite La_0.5_Ba_0.5_CoO_3−δ_ and the layered double perovskite LaBaCo_2_O_5+δ_ materials from this work compared to a single perovskite BaCo_0.4_Fe_0.4_Zr_0.1_Y_0.1_O_3−δ_ and a layered double perovskite La_0.2_Gd_0.8_BaCo_2_O_5+δ_ cathodes materials from the literature [3,16]. The lines represent the slope used to calculate E_a_.

Figure 9 compares the performance of both LaBaCo_2_O_5+δ_ and La_0.5_Ba_0.5_CoO_3−δ_ materials with the two best PCFC cathode materials reported in the literature: the single perovskite BaCo_0.4_Fe_0.4_Zr_0.1_Y_0.1_O_3−δ_ (BCFZY) [3] and the layered double perovskite La_0.2_Gd_0.8_BaCo_2_O_5+δ_ (LGBC) [16]. The comparison is carried out by taking literature data measured in the same configuration (four-electrode measurements of electrolyte supported symmetric cells), rather than complete fuel cells. One well known issue with symmetric cell measurements involving PCFC electrolytes in oxidizing atmospheres is the influence of the parasitic *p*-type conductivity of the electrolyte itself [16] on the apparent measured cathode ASR (especially at high temperatures) [42]. This parasitic *p*-type electronic conductivity leads to an overestimation of the performance of the electrode and makes the interpretation of the data more complex. Thus, it is not recommended to compare cathode ASR results obtained from complete fuel cells to the results obtained from symmetric cell studies. Symmetric cell comparisons, however, are likely to be reasonable if the investigations employ similar electrolyte compositions and thicknesses. Based on such symmetric cell comparisons, the performance of both LaBaCo_2_O_5+δ_ and La_0.5_Ba_0.5_CoO_3−δ_ materials are comparable to LGBC and BCFZY, with even better performance at temperatures above 500 °C. These comparisons underscore the high potential of both LaBaCo_2_O_5+δ_ and La_0.5_Ba_0.5_CoO_3−δ_ materials as PCFC cathodes.

Activation energies are summarized in Figure 9. Both LaBaCo_2_O_5+δ_ and La_0.5_Ba_0.5_CoO_3−δ_ materials have higher activation energies than LGBC and BCFZY, which suggests differences in the electrode electrochemical mechanism. A preliminary assignment of the electrochemical mechanism can be suggested by looking at the temperature dependence of the deconvoluted electrochemical processes shown in Figure 4. The low frequency process is hardly dependent on the temperature which may be assigned to the oxygen adsorption/dissociation processes at all, while the intermediate frequency process may be assigned to charge transfer processes due to the higher temperature dependency of this process. This is consistent with previous works in the literature on materials with similar perovskite structure [16,38].

In addition to this, it is well-known that the microstructure of cathode materials plays a crucial role for the electrochemical properties. Adler et al. [43] published a detailed study on the electrode kinetics of porous mixed-conducting oxygen electrodes based on oxide-ion conducting electrolyte, using a continuum modeling to analyze the oxygen reduction reaction. Furthermore, Strandbakke et al. [44] showed that pre-exponential values of the Arrhenius plot are indicative of the microstructure impact in the electrochemical performance. A similar microstructure for both LaBaCo_2_O_5+δ_ and La_0.5_Ba_0.5_CoO_3−δ_ materials can be seen in Figure S1 and can also be confirmed with the similar pre-exponential values calculated from Figure 9 (13.55 for La_0.5_Ba_0.5_CoO_3−δ_ and 14.75 for LaBaCo_2_O_5+δ_). However, experiments at different pO_2_ and pH_2_O and more detailed microstructure studies (determination of the tortuosity, surface area, etc.) are necessary to successfully assign the electrochemical processes and to correlate them with the electrode microstructure [39].

Table 1 summarizes the structure, Ba content, performance, oxygen content and electrical conductivities of LaBaCo_2_O_5+δ_ and La_0.5_Ba_0.5_CoO_3−δ_. The table also includes data for BCFZY [3,13] and LGBC [16,43]. Therefore, due to the similar microstructure discussed previously we attempt a correlation between all these parameters to help rationalize the performance differences between these various cathode materials.

### 4.2. Correlation between ASR, Electrical Conductivity, Basicity and Oxygen Content

**Figure 10:**
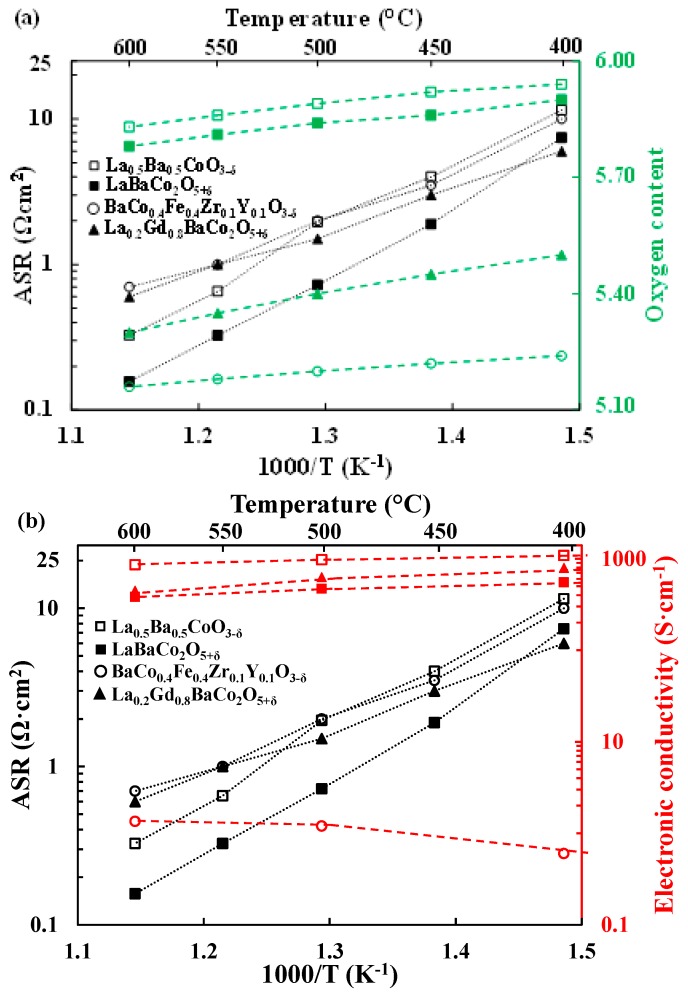
Area Specific Resistances (ASR, Ω·cm^2^) (black symbols) and oxygen content (green symbols) variation (**a**) together with electrical conductivity (red symbols) (**b**) as a function of temperature for the single perovskite La_0.5_Ba_0.5_CoO_3−δ_ and the layered double perovskite LaBaCo_2_O_5+δ_ materials compared to the single perovskite BaCo_0.4_Fe_0.4_Zr_0.1_Y_0.1_O_3−δ_ and the layered double perovskite La_0.2_Gd_0.8_BaCo_2_O_5+δ_ cathodes materials from the literature [3,13,16,45]. Oxygen contents for both materials are normalized as O_6_. The lines are guides for the eye.

The ASR and the oxygen content as a function of temperature for both La_0.5_Ba_0.5_CoO_3−δ_ and LaBaCo_2_O_5+δ_ materials are shown in Figure 10a. Likewise, the ASR and the electrical conductivity vs. temperature are plotted in Figure 10b. Data for the single perovskite BCFZY [3,13] and the layered double perovskite LGBC [16,45] materials extracted from the literature are added for comparison. Two observations are evident from these two figures: (1) a lower oxygen deficiency of La_0.5_Ba_0.5_CoO_3−δ_ and LaBaCo_2_O_5+δ_ compared to BCFZY and LGBC; (2) a significantly lower (nearly two orders of magnitude) electrical conductivity for BCFZY.

The lower performance of La_0.5_Ba_0.5_CoO_3−δ_ in comparison to LaBaCo_2_O_5+δ_ at lower temperatures can be explained by the lower oxygen vacancy concentration in La_0.5_Ba_0.5_CoO_3−δ_ as shown in Figure 10. The enhanced low-temperature oxygen vacancy concentrations for both LGBC and BCFZY materials appear to be consistent with their higher low-temperature cathode performance.

Protonation of oxygen vacancies generally increases with oxide basicity [13,14]. All else being equal, it is therefore expected that more basic materials will have higher performance levels as PCFC cathodes. In addition, higher oxygen vacancy concentration should drive higher protonation according to Le Chatelier’s principle. Zohourian et al. [13] and Strandbakke et al. [16] have measured the hydration level in BCFZY and LGBC. Despite their high level of oxygen vacancies both materials are only able to hydrolyze 1% of the available oxygen vacancies at 400 °C. Because of its higher basicity, a greater fraction of vacancies is expected to be hydrolyzed in BCFZY. Nevertheless, Figure 9 and Table 1 show that at 400 °C the performance of the layered double perovskite material is better than the materials with single perovskite structure. One possible explanation of this difference could be that A-site cation ordering enhances protonation due to the different local environments of the oxygen vacancies in the two crystal structures. The similar performance of LaBaCo_2_O_5+δ_ and LGBC can also be explained by the higher basicity of the A-site cations in LaBaCo_2_O_5+δ_.

A-site cation ordering in LaBaCo_2_O_5+δ_ leads to higher performance [30] due to the higher oxygen vacancy concentration as shown in Figure 10a. The higher oxygen vacancy concentration and therefore higher degree of protonation could rationalize the higher performance of LaBaCo_2_O_5+δ_ compared to La_0.5_Ba_0.5_CoO_3−δ_ Furthermore, both LaBaCo_2_O_5+δ_ and La_0.5_Ba_0.5_CoO_3−δ_ as well as BCFZY have been shown to be very good oxide ion conductors [26,27,28,46]. Therefore, the main difference in performance between BCFZY and both LaBaCo_2_O_5+δ_ and La_0.5_Ba_0.5_CoO_3−δ_ materials may arise from the differences in electrical conductivity as shown in Figure 10b in addition to a difference in proton conductivity.

Measurements in low pO_2_ conditions have been proposed in order to eliminate the presence of oxide-ions and isolate the proton conduction contribution when examining prospective PCFC cathodes [16]. As shown in Figure 5, a decrease in ASR and E_a_ for both LaBaCo_2_O_5+δ_ and La_0.5_Ba_0.5_CoO_3−δ_ is observed in humidified inert atmosphere (pO_2_ = 10^−4^ atm). This decrease may suggest the presence of proton conductivity in the material, and is similar to what was observed for LGBC by Strandbakke et al. [16].

### 4.3. Chemical Stability of the Two Polymorphs

Previous studies of the oxygen non-stoichiometry of the two polymorphs have suggested that La_0.5_Ba_0.5_CoO_3−δ_ is the most stable polymorph at oxidation conditions and low temperature [25]. The coulometric titration data (Figure 7) confirms that the oxygen content of LaBaCo_2_O_5+δ_ is lower than La_0.5_Ba_0.5_CoO_3−δ_ independent of temperature and oxygen partial pressure. The lower oxygen partial pressure at the decomposition suggests that La_0.5_Ba_0.5_CoO_3−δ_ is more stable than LaBaCo_2_O_5+δ_ also under reducing conditions.

It has previously been shown that La_0.5_Ba_0.5_CoO_3−δ_ can be transformed to LaBaCo_2_O_5+δ_ by annealing in N_2_ at 1100 °C [25], while we demonstrated in this work that the transverse phase transition is observed in air close to 1100 °C. Based on these two observations LaBaCo_2_O_5+δ_ is the stable polymorph at inert conditions and LaBaCo_2_O_5+δ_ the most stable in air close to 1100 °C. Evidence for a phase transition between the two polymorphs could not be obtained by coulometric titration up to 800 °C (Figure 7). The phase transition is however reconstructive in nature and only occurs above a critical temperature for sufficient cation mobility. It is therefore likely that true equilibrium between the two phases could not be obtained during the coulometric titration before decomposition. Additional measurements are therefore required to determine accurately the relative stability of the two polymorphs and the T-pO_2_ dependence of the phase transition between the two polymorphs.

## 5. Conclusions

LaBaCo_2_O_5+δ_ and La_0.5_Ba_0.5_CoO_3−δ_ materials have been tested as cathodes for protonic ceramic fuel cells and both exhibit good stabilities. The A-site cation ordered LaBaCo_2_O_5+δ_ material possesses better performance than the A-site cation disordered La_0.5_Ba_0.5_CoO_3−δ_ materials with ASR values as low as 7.4 and 0.16 Ω·cm^2^ at 400 and 600 °C respectively. Both LaBaCo_2_O_5+δ_ and La_0.5_Ba_0.5_CoO_3−δ_ demonstrate competitive performance with the best state-of-the-art cathode materials BaCo_0.4_Fe_0.4_Zr_0.1_Y_0.1_O_3−δ_ and La_0.2_Gd_0.8_BaCo_2_O_5+δ_. Oxygen vacancy concentration, electrical conductivity, crystal structure and basicity are shown to be key, interconnected parameters that govern PCFC cathode performance. The A-site cation ordered LaBaCo_2_O_5+δ_ material exhibits better performance at low temperature than the A-site cation disordered La_0.5_Ba_0.5_CoO_3−δ_ material because it retains higher oxygen vacancy concentration at low temperatures. In addition, A-site cation ordering is hypothesized to increase the basicity of the oxygen vacancies, making them more likely to hydrate. The similar low-temperature performance of A-site cation ordered LaBaCo_2_O_5+δ_ material vs. La_0.2_Gd_0.8_BaCo_2_O_5+δ_ (LGBC) may be explained by an increase of the basicity in LaBaCo_2_O_5+δ_. As the temperature increases, the A-site cation ordered LaBaCo_2_O_5+δ_ material shows better relative performance compared to the A-site cation disordered La_0.5_Ba_0.5_CoO_3−δ_ due to the increase in oxygen vacancy concentration. This work shows the importance of understanding and controlling crystal structure, basicity, oxygen vacancy concentration and electrical conductivity in order to improve PCFC cathode materials while keeping an eye on the chemical stability of the material.

## Figures and Tables

**Table 1:** Structure, Ba per mol, electrochemical performance at 400, 600 °C and in 3% H_2_O air, oxygen content and electrical conductivity at 400 °C and 600 °C in air for A-site cation disordered La_0.5_Ba_0.5_CoO_3−δ_ and A-site cation ordered LaBaCo_2_O_5+δ_ materials compared to the single perovskite BaCo_0.4_Fe_0.4_Zr_0.1_Y_0.1_O_3−δ_ (BCFZY) and the layered double perovskite La_0.2_Gd_0.8_BaCo_2_O_6−δ_ (LGBC) extracted from the literature.

Ba per mol	Structure	Material	Performance (Ω·cm^2^) at 400 °C and 3% H_2_O in Air	Performance (Ω·cm^2^) at 600 °C and 3% H_2_O in Air	Oxygen Content at 400 °C in Air	Oxygen Content at 600 °C in Air	Electrical Conductivity at 400 °C in Air (S·cm^−1^)	Electrical Conductivity at 600 °C in Air (S·cm^−1^)
1	Single perovskite	BaCo_0.4_Fe_0.4_Zr_0.1_Y_0.1_O_3−δ_ (BCFZY)	23.03	0.703	2.6713	2.5813	0.603	1.353
0.5		La_0.5_Ba_0.5_CoO_3−δ_	11.5	0.33	2.97	2.92	1085.0	857.0
	Layered double perovskite	LaBaCo_2_O_5+δ_	7.4	0.16	2.95	2.89	554.0	384.0
		La_0.2_Gd_0.8_BaCo_2_O_6−δ_ (LGBC)	6.016	0.616	2.7516	2.6516	<795 (GdBaCo_2_O_5+δ_)^5^	<447 (GdBaCo_2_O_5+δ_)^5^

## Supplementary Materials

The following are available online at http://www.mdpi.com/1996-1944/11/2/196/s1, Figure S1: Scanning electron micrographs of fractured cross sections from tested electrolyte supported symmetric cells of for both La_0.5_Ba_0.5_CoO_3−δ_ and LaBaCo_2_O_5+δ_. Figure S2: X-ray diffraction patterns showing the reactivity of BaZr_0.9_Y_0.1_O_3−δ_ with La_0.5_Ba_0.5_CoO_3−δ_ at 1000 °C, 1100 °C and 1200 °C for 72 h. Impurities, shown by asterisks at 1200 °C, are identified as LaCoO_3_ and BaCoO_3_. Table S1: Area specific resistances (R) and pseudo-capacitance (C) values from the fitting of the electrochemical model for both La_0.5_Ba_0.5_CoO_3−δ_ and LaBaCo_2_O_5+δ_.
