# Supplementary material for: Effect of Cation Ordering on the Performance and Chemical Stability of Layered Double Perovskite Cathodes
Source: Materials (Basel). 2018 Jan 26;11(2):196. doi: 10.3390/ma11020196 (PMC5848893; doi:10.3390/ma11020196)
Supplement: Supplementary file 1 [file materials-11-00196-s001.pdf]

Supplementary

Effect of cation ordering on the performance and chemical stability of layered double perovskite cathodes

Carlos Bernuy-Lopez[(a)1*], Laura Rioja-Monllor[(a)], Takashi Nakamura[(b)], Sandrine Ricote[(c)], Ryan O'Hayre[(d)], Koji Amezawa[(b)], Mari-Ann Einarsrud[(a)], Tor Grande[(a)]

[(a)] Department of Material Science and Engineering, NTNU Norwegian University of Science and Technology, Trondheim NO-7491, Norway.
[(b)] Institute of Multidisciplinary Research for Advanced Materials, Tohoku University, 2-1-1 Katahira Aoba-ku, Sendai 980–8577, Japan.
[(c)] Department of Mechanical Engineering, Colorado School of Mines, Golden, CO 80401, USA
[(d)] Department of Metallurgical and Materials Engineering, Colorado School of Mines, 1500 Illinois St. Golden, CO 80401, USA.
* Corresponding Author: carlos.bernuy-lopez@sandvik.com;
[1]Current address: Sandvik Materials and Technology, R&D, Sandviken. Sweden

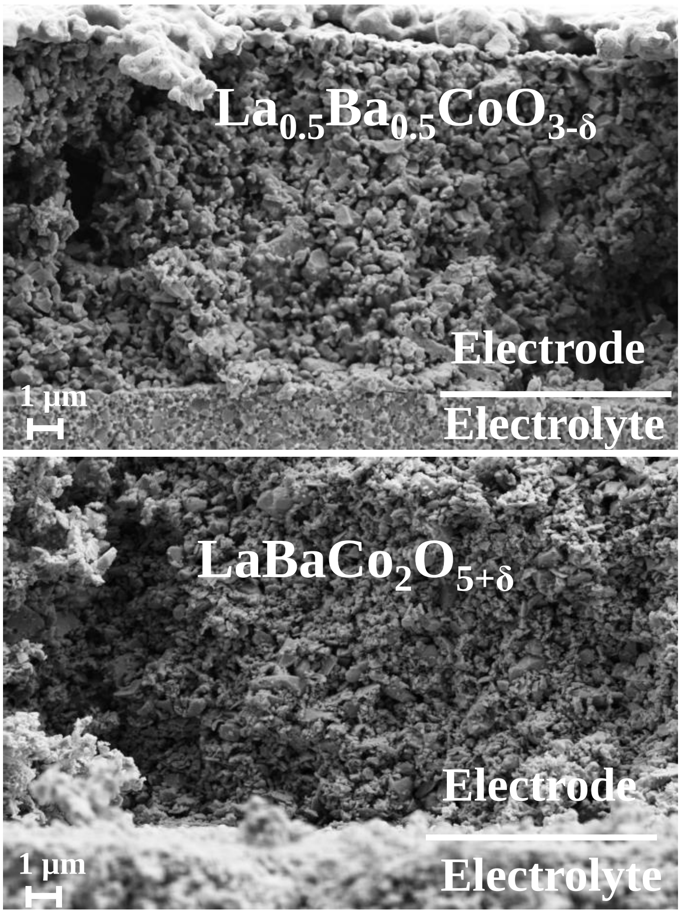

Figure S1. Scanning electron micrographs of fractured cross sections from tested electrolyte supported symmetric cells of for both $La_{0.5}Ba_{0.5}CoO_{3-\delta}$ and $LaBaCo_2O_{5+\delta}$.

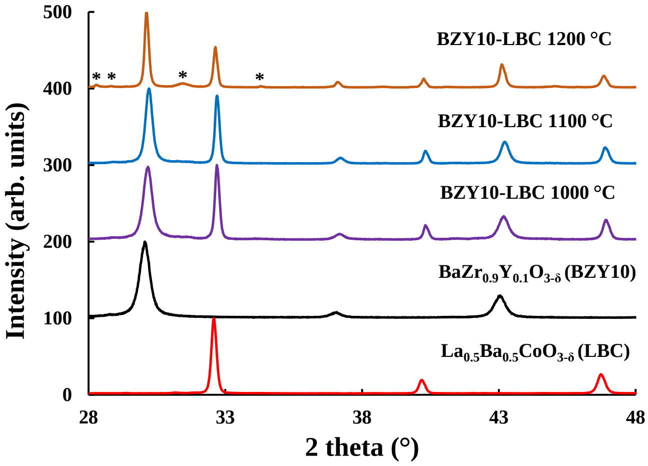

Figure S2. X-Ray diffraction patterns showing the reactivity of $BaZr_{0.9}Y_{0.1}O_{3-\delta}$ with $La_{0.5}Ba_{0.5}CoO_{3-\delta}$ at 1000 °C, 1100 °C and 1200 °C for 72 h. Impurities, shown by asterisks at 1200 °C, are identified as $LaCoO_3$ and $BaCoO_3$.

Table S1. Area specific resistances (R) and pseudo-capacitance (C) values from the fitting of the electrochemical model for both $La_{0.5}Ba_{0.5}CoO_{3-\delta}$ and $LaBaCo_2O_{5+\delta}$.

**$La_{0.5}Ba_{0.5}CoO_{3-\delta}$**							
T (°C)	R_{BZY10_1} (Ω cm²)	R_{BZY10_2} (Ω cm²)	CPE_{BZY10_2} (F/cm²)	R_{SP_1} (Ω cm²)	CPE_{SP_1} (F/cm²)	R_{SP_2} (Ω cm²)	CPE_{SP_2} (F/cm²)
400	24.58	170.66	8.82E-10	22.11	2.91E-03	0.87	7.56E-02
450	8.81	102.49	1.90E-09	6.12	9.35E-04	1.88	1.72E-01
500	17.17	56.18	1.62E-09	3.06	6.11E-04	0.85	1.54E+00
550	14.84	36.31	3.37E-11	0.43	1.64E-03	0.87	2.27E-01
600	20.32	16.11	6.19E-10	0.15	1.89E-03	0.50	1.53E-01

**$LaBaCo_2O_{5+\delta}$**							
T (°C)	R_{BZY10_1} (Ω cm²)	R_{BZY10_2} (Ω cm²)	CPE_{BZY10_1} (F/cm²)	R_{LP_1} (Ω cm²)	CPE_{LP_1} (F/cm²)	R_{LP_2} (Ω cm²)	CPE_{LP_2} (F/cm²)
400	10.86	32.82	5.27E-09	12.55	2.20E-04	2.31	1.89E-03
450	4.00	17.92	1.43E-07	3.27	2.46E-04	0.52	2.62E-01
500	12.85	1.08	7.73E-07	0.99	2.77E-04	0.43	2.56E-01
550	0.18	9.12	1.56E-05	0.31	3.81E-04	0.34	3.03E-01
600	6.52	0.15	3.59E-06	0.08	1.62E-03	0.23	4.87E-01
